# Fluctuations in Human Corticospinal Activity Prior to Grasp

**DOI:** 10.3389/fnsys.2019.00077

**Published:** 2019-12-20

**Authors:** Nishant Rao, Pranav J. Parikh

**Affiliations:** Center for Neuromotor and Biomechanics Research, Department of Health and Human Performance, University of Houston, Houston, TX, United States

**Keywords:** noise, preparation, TMS, motor cortex, force, CSE, variability

## Abstract

Neuronal firing rate variability prior to movement onset contributes to trial-to-trial variability in primate behavior. However, in humans, whether similar mechanisms contribute to trial-to-trial behavioral variability remains unknown. We investigated the time-course of trial-to-trial variability in corticospinal excitability (CSE) using transcranial magnetic stimulation (TMS) during a self-paced reach-to-grasp task. We hypothesized that CSE variability will be modulated prior to the initiation of reach and that such a modulation would explain trial-to-trial behavioral variability. Able-bodied individuals were visually cued to plan their grip force before exertion of either 30% or 5% of their maximum pinch force capacity on an object. TMS was delivered at six time points (0.5, 0.75, 1, 1.1, 1.2, and 1.3 s) following a visual cue that instructed the force level. We first modeled the relation between CSE magnitude and its variability at rest (*n* = 12) to study the component of CSE variability pertaining to the task but not related to changes in CSE magnitude (*n* = 12). We found an increase in CSE variability from 1.2 to 1.3 s following the visual cue at 30% but not at 5% of force. This effect was temporally dissociated from the decrease in CSE magnitude that was observed from 0.5 to 0.75 s following the cue. Importantly, the increase in CSE variability explained at least ∼40% of inter-individual differences in trial-to-trial variability in time to peak force rate. These results were found to be repeatable across studies and robust to different analysis methods. Our findings suggest that the neural mechanisms underlying modulation in CSE variability and CSE magnitude are distinct. Notably, the extent of modulation in variability in corticospinal system prior to grasp within individuals may explain their trial-to-trial behavioral variability.

## Introduction

Trial-to-trial variability is an inherent feature of motor behavior (Stein et al., 2005; Faisal et al., 2008). Intertrial variability in motor output reflects the presence of stochastic noise in the sensorimotor system and may interfere with one’s ability to perform a given movement consistently (Harris and Wolpert, 1998; Slifkin and Newell, 1999; Stein et al., 2005; Faisal et al., 2008). Another perspective suggests that the intertrial motor output variability provides the sensorimotor system an ability to explore the motor workspace for optimizing motor learning (Tumer and Brainard, 2007; Wu et al., 2014).

Several studies have found central correlates of variability in kinematic or kinetic features of motor output (Osborne et al., 2005; Churchland et al., 2006a; Fox et al., 2007; Hohl et al., 2013; Chaisanguanthum et al., 2014; Lisberger and Medina, 2015; Mizuguchi et al., 2016; Haar et al., 2017). In monkeys, variable activity of sensory neuronal populations within extrastriate MT region explained variability in execution of smooth-pursuit eye movement (Hohl et al., 2013). Firing rates of neurons within primate primary motor (M1) and premotor cortices prior to movement onset explained intertrial variability in peak reach velocity (Churchland et al., 2006a). In humans, variation in fMRI responses within inferior parietal lobule observed during motor execution has been shown to explain differences in intertrial variability in reach kinematics across individuals (Haar et al., 2017). However, in humans, the contribution of central mechanisms engaged prior to movement onset to variability in motor output remains to be known.

Motor evoked potentials (MEP) elicited non-invasively using transcranial magnetic stimulation (TMS) can provide information regarding the neural mechanisms at cortical, subcortical, and spinal levels, i.e., corticospinal excitability (CSE), during various phases of a task (Bestmann and Krakauer, 2015). For instance, CSE is modulated by activity within inferior parietal lobule and caudal intraparietal sulcus while preparing a contralateral reach (Koch et al., 2008). The modulation in intertrial variability in CSE assessed prior to movement onset has been shown to encode value-based decision-making processes and differentiate fast versus slow reaction time responses (Klein-Flugge et al., 2013). These findings suggest that the temporal unfolding of CSE variability from trial-to-trial may provide information regarding task-related central processes that may be related to and/or explain an observed behavior. However, whether intertrial CSE variability assessed prior to movement onset explains intertrial variability in motor output remains to be known. In the current study, we investigated the time course of CSE variability during a self-paced, isometric grip force production task in healthy young adults and studied whether the modulation in CSE variability explained differences in trial-to-trial variability in the application of grip force across individuals. We studied a grasping task because our earlier work found CSE to be a sensitive measure to investigate neural mechanisms prior to grasp (Parikh et al., 2014). Subjects were instructed to first reach for an instrumented object, grasp it, and apply grip force. They were cued to exert either 30% or 5% of the maximal pinch force during the task. We delivered TMS pulses over M1 at different time points following the cue but before the onset of reach to assess the temporal unfolding of CSE variability. Intertrial variability in CSE assessed in this manner may be related to intrinsic changes in MEP amplitude, a phenomenon that has been studied before (Stein et al., 2005; Darling et al., 2006; Faisal et al., 2008; Bestmann and Krakauer, 2015). Therefore, we modeled a relation between CSE variability and its amplitude in absence of a task during a separate session (Darling et al., 2006; Klein-Flugge et al., 2013). This allowed us to study the component of CSE variability that was beyond the intrinsic changes in CSE magnitude. We hypothesized that CSE variability would be modulated prior to the onset of reach. As CSE variability may represent neural variability and as the latter is known to explain behavioral variability in primates (Churchland et al., 2006a), we expected that individuals with greater modulation in CSE variability would exhibit a greater intertrial variability in their grip force application. We expected differences in these findings for the two force levels because the neural activity might be dependent on the magnitude of force (Dettmers et al., 1996; Ehrsson et al., 2001; Hendrix et al., 2009; Perez and Cohen, 2009; Parikh et al., 2014).

## Materials and Methods

### Subjects

Thirteen young, healthy, right-handed subjects (Oldfield, 1971) aged between 18 and 36 years (mean ± SD: 25.30 ± 3.59 years; four females) provided written informed consent to participate in this study. Subjects eligible for the protocol had a normal or corrected-to-normal vision, no upper limb injury, and no history of neurological diseases or musculoskeletal disorders. They were screened for potential risks or adverse reactions to TMS using the TMS Adult Safety Screen questionnaire (Keel et al., 2001; Rossi et al., 2009). The study was approved by the Institutional Review Board of the University of Houston.

### Experimental Apparatus

**FIGURE 1:**
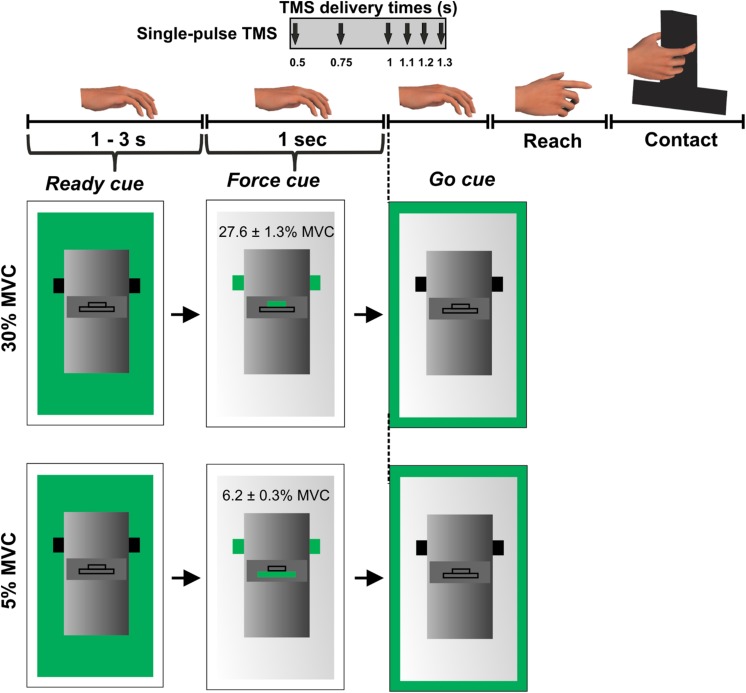
Experimental protocol. Figure adapted from Parikh et al. (2014).

Subjects were instructed to grasp a custom-designed inverted T-shaped grip device using their index finger and thumb. Two six-dimensional force/torque transducers (Nano-25; ATI Industrial Automation, Garner, NC, United States), mounted on the grip device, measured the forces and moments exerted by their index finger and thumb on the object (Figure 1). Force data were acquired using a 12-bit analog-to-digital converter board (sampling frequency 1 kHz; model PCI-6225; National Instruments, Austin, TX, United States).

### Electromyography (EMG)

We recorded muscle activity from first dorsal interosseous (FDI), abductor policis brevis (APB), and abductor digiti minimi (ADM) using differential surface electrodes (band-pass filter with a cut-off frequency range of 20–450 Hz (Grecco et al., 2016; Guerra et al., 2016; Torrecillos et al., 2019); Delsys Bagnoli EMG System, Boston, MA, United States). EMG data were sampled at 5 kHz using CED data acquisition board (Micro1401, Cambridge, United Kingdom). Both force and EMG data were analyzed using custom-made MATLAB script (R2016b; Mathworks, Natick, MA, United States).

### Transcranial Magnetic Stimulation

Single-pulse TMS was used to assess CSE during the experiment (Parikh et al., 2014; Davare et al., 2019; Goel et al., 2019; Rao et al., 2019). We first estimated the resting motor threshold (rMT) by delivering suprathreshold single monophasic TMS pulses (Magstim 200, Whitland, United Kingdom) with the TMS coil held tangential to the scalp and perpendicular to the presumed direction of the central sulcus, 45° from the midsagittal line, with the handle pointing backward, inducing current in the posteroanterior direction. The coil position was adjusted to optimize the motor-evoked potential (MEP) in all recorded muscles. Following this procedure, the rMT was estimated as the minimum TMS-intensity to elicit motor evoked potential (MEP) with an amplitude of ∼50 μV (peak-to-peak) for at least 5 of the 10 consecutive trials in the FDI muscle (Klein-Flugge et al., 2013; Parikh et al., 2014; Rossini et al., 2015; Rao et al., 2019). The TMS coil was stabilized using a coil holder mounted on the TMS chair (Rogue Research). The TMS coil was traced on the subject’s scalp using a surgical marker pen. The coil location was regularly checked for any displacement that might have occurred during a session. The average rMT across subjects (mean ± SE) was 41 ± 3% of the maximum stimulator output.

### Experimental Design

Eleven of thirteen subjects participated in two experiments performed at least 24 h apart. Two subjects were able to participate in one of the two experiments. The two experimental sessions were counterbalanced across subjects.

#### Experiment 1 (At Rest; *n* = 12)

We established a relation between the variability in MEP and its amplitude at rest. We delivered single pulse TMS at the following TMS intensities: 0.9, 1, 1.1, 1.2, 1.3, 1.4, 1.5, 1.6, and 1.7 times the rMT (Darling et al., 2006) with 10 consecutive pulses delivered at each intensity in a randomized order. Subjects neither performed a task nor received a stimulus except TMS.

#### Experiment 2 (the Force Task; *n* = 12)

During this session, we asked subjects to perform an isometric force production task using their index finger and thumb of the right hand. The distance between the grip device and the hand was ∼30 cm at the beginning of each trial. Subjects were instructed to reach for the grip device, grasp the device at the same locations, and exert grip force to match a target on computer monitor using their index finger and thumb (Figure 1).

We introduced two different force levels (30% and 5% of maximal pinch force) to investigate modulation in MEP variability at different force magnitudes. Self-selection of digit contact points during object grasping would require subjects to plan digit placement (Davare et al., 2019), making it challenging to isolate the digit force planning component embedded in the reach-to-grasp task. To rule out differential planning of digit position from trial-to-trial, we instructed subjects to grasp the device at marked locations on every trial. This location was denoted by a black tape attached on the front panel of the grip device (Parikh et al., 2014). A computer monitor placed behind the device displayed three sequential visual cues on every trial: “ready,” “force” and “go.” The “ready” cue signaled the beginning of a trial. The “force” cue informed the subject about whether the upcoming force task required 5% or 30% of grip force application. Finally, the “go” cue instructed subjects to initiate reach and perform the force production task. The “ready” and “force” cues were separated by a randomly varying interval between 1 and 3 s while “force” and “go” cues were separated by 1 s (Figure 1). Subjects were instructed to apply grip force to reach the target (displayed on the computer monitor during the “force” cue presentation) at a self-selected speed and maintain that force for 3 s using their right hand. Visual feedback of subject’s grip force was provided during each trial. Subjects practiced the force production task to get familiarized with the experimental task before the session. At the beginning of the session, we measured maximum voluntary contraction (MVC) for each subject by asking them to apply maximum pinch force only using right thumb and index finger. We selected the largest force of three MVC trials to set the force target. The variability in MEP is sensitive to the magnitude of background EMG activity (Darling et al., 2006). Thus, during all reach and grasp trials, subjects were instructed to maintain their muscles in a relaxed state until the “go” cue. This setup allows comparable EMG activity across trials and minimizes the confounding effect of background muscle activity on MEP variability.

While subjects performed the *force task*, single TMS pulses at 120% of rMT were delivered to the scalp location for FDI marked earlier at 1 of the 6 latencies in a randomized order: 0.5, 0.75, 1 (“go”), 1.1, 1.2, or 1.3 s following the “force” cue but prior to the onset of reach (Figure 1). Each subject performed 15 trials per TMS time point and force level in a randomized sequence. As there were six TMS time points across two force levels, subjects performed 180 trials across four blocks with 45 trials per block and with ∼5 min of rest between the blocks. The minimum time interval between successive TMS pulses was ∼15 s. For this experimental setup, our earlier study has shown that the reach is not initiated at least until 0.4 s after the “go” cue (i.e., 1.4 s following the “force” cue) (Parikh et al., 2014).

### Data Processing and Statistical Analysis

#### Behavioral Variability in the Application of Force

We focused our analysis on the peak force rate (PFR) application because it is known to be influenced by planning-related mechanisms (Johansson and Westling, 1988; Gordon et al., 1993). In presence of visual feedback, as in this study, peak force rate is also influenced by online adjustments of grip force. We analyzed magnitude and time to peak force rate (Time_PFR_) to assess behavioral variability, as previously reported by Flanagan and Beltzner (2000) and Poston et al. (2008). To compute the rate of grip force application, we first smoothed the grip force signal through a zero-phase lag, fourth order, low-pass Butterworth filter (cutoff frequency: 14 Hz) followed by calculating its first derivative with respect to the trial time (Flanagan and Beltzner, 2000). Separate analyses were then conducted for peak force rate (PFR) and Time_PFR_. Intertrial variability in these measures were assessed by calculating their standard deviation (SD) around the mean value for each TMS delivery time point and force level. We performed repeated measures analysis of variance (rmANOVA) with within-subject factors such as TMS (0.5, 0.75, 1, 1.1, 1.2, and 1.3 s) and FORCE (5% and 30% of force).

#### Intertrial Variability in Corticospinal Excitability (CSE)

Motor evoked potentials elicited using single TMS pulses were recorded to estimate the CSE (Parikh and Santello, 2017) during both sessions (Klein-Flugge et al., 2013; Parikh et al., 2014; Parikh and Santello, 2017). MEPs were also identified with pre-stimulus EMG contamination if the signal within 100 ms before TMS contained a peak-to-peak amplitude ≥ 0.1 mV (Klein-Flugge et al., 2013) and were removed from subsequent analysis (∼2% of trials per subject). For *experiment* 1, the data were divided into nine bins (10 trials for each stimulus intensity) per subject representing MEPs at a given intensity (Klein-Flugge et al., 2013). Bins with more than five trials were included if the average MEP exceeded 0.1 mV (Klein-Flugge et al., 2013). Bins with average MEPs exceeding the average MEP amplitude from all the bins by three standard deviations were identified and excluded from further analysis (Klein-Flugge et al., 2013). For *experiment 2*, MEPs with pre-EMG contamination (peak-to-peak signal ≥ 0.1 mV within 100 ms before TMS pulse; ∼2% trials per subject) and MEP measuring < 0.1 mV were discarded from the subsequent analysis (∼8% trials per subject), thus matching the criteria used for *experiment 1.* Overall, ∼10% of trials were excluded during data processing across FDI, APB, and ADM muscles and time points. The coefficient of variation (CV = SD/mean) of MEP was used to quantify the intertrial variability in MEP (Klein-Flugge et al., 2013). MEP variability depends on the proportion of neurons in the motoneuron pool which are not consistently recruited by the stimulus (Kiers et al., 1993). At lower TMS intensities, a smaller proportion of neurons are consistently recruited, thus resulting in higher MEP variability. Conversely, at higher TMS intensities a larger proportion of neurons are consistently recruited leading to lower MEP variability. Consistent with this argument, several studies have found an inverse relationship between the coefficient of variation (CV) of MEP responses and MEP amplitude (Kiers et al., 1993; Devanne et al., 1997; Capaday et al., 1999; Darling et al., 2006; Klein-Flugge et al., 2013).

From *experiment 1*, we modeled a relation between MEP amplitude and its variability at rest using individual data points from all subjects (Darling et al., 2006; Klein-Flugge et al., 2013). The function characterizing this relationship was identified as the logarithmic curve of the form:

(1) CV=a×log⁡(amplitude)+b

The parameters *a* (slope) and *b* (intercept) were identified using the *modelfun* function in MATLAB (Mathworks, Natick, MA, United States). These parameters were identified separately for three muscles (FDI, APB, and ADM). We also assessed the residuals for the logarithmic fits for each muscle.

From *experiment 2*, intertrial variability in MEPs (observed CV or CV_OBS_) was assessed by calculating the CV of MEPs (Klein-Flugge et al., 2013) for every TMS time point separately for each force level. The logarithmic model obtained from *experiment 1* was used to predict the CV of MEP (CV_PRED_) that was primarily due to intrinsic changes in MEP amplitude. Such a model has been found to robustly estimate CV_PRED_ for any intercept parameter and for the slope parameter within the range of −0.5 to infinity (Klein-Flugge et al., 2013). This predicted variability was then subtracted from the observed variability in CSE from *experiment 2* (CV_DIFF_ of MEP = CV_OBS_ − CV_PRED_). The observed variability is a combination of intrinsic variability and task-related variability (Fox et al., 2007; Klein-Flugge et al., 2013). The resultant MEP variability (CV_DIFF_) or its modulation may represent task-related mechanisms (Klein-Flugge et al., 2013). For CV_PRED_ and CV_DIFF_ of MEP, we performed separate rmANOVA (α = 0.05) with within-subject factors such as FORCE (two levels: 5% and 30% of force) and TMS (six levels: 0.5, 0.75, 1, 1.1, 1.2, and 1.3 s) for three muscles (FDI, APB, ADM).

To assess the repeatability of our findings, we performed this analysis on a dataset from nine additional subjects. These subjects performed the force production task at either 10% of force or 1 N force under similar experimental paradigm (Parikh et al., 2014). As *experiment 1* was not conducted in the earlier study, we used the logarithmic model obtained using data points from all subjects in the current study. This logarithmic model resulted in the same results in *experiment 2*. These findings were presented earlier at the annual meeting of the Society for Neuroscience (Rao and Parikh, 2017) and are summarized in the section “Results.”

To assess the robustness of the analytical approach, we analyzed data without using a lower bound cut-off criterion for MEP amplitude and without using a bin-based cut-off criterion. Furthermore, the results may be sensitive to parameters obtained by fitting a logarithmic model on data points from all subjects (i.e., a group-level model; *experiment 1* description above) versus fitting a separate model on data points from each subject (i.e., subject-level models). For each subject, a subject-level logarithmic model was used to calculate CV_PRED_ for each subject and the resulting CV_DIFF_ showed similar findings in *experiment 2* (see section “Results”).

To investigate whether MEP variability assessed prior to reach onset explained inter-individual differences in behavioral variability, we performed separate Pearson product-moment correlation analysis between the CV_DIFF_ of MEP and the behavioral measures, i.e., SD of PFR and Time_PFR_ and CoP ellipse area. We also performed the correlation analysis using standard deviation as a parameter to confirm that the findings are not sensitive to the measure of MEP variability (CV versus SD). First, we computed the predicted value of SD from CV_PRED_ (SD_PRED_ = CV_PRED_ × mean MEP amplitude). Next, we computed the inter-trial SD from the force task (experiment 2) to obtain SD_OBS_. Finally, we obtained the SD_DIFF_ of MEP by subtracting SD_PRED_ from SD_OBS_ (SD_DIFF_ = SD_OBS_ − SD_PRED_).

#### EMG Analysis

We quantified the modulation in FDI and APB muscles involved in the production of grip force when subjects applied 30% and 5% of force on the object. For this purpose, we calculated the root mean square (RMS) value of the EMG signal for a 1.5 s segment during steady force production at 5% and 30% of force separately for FDI and APB (Zhang et al., 2017; Hu et al., 2018). For data from each muscle, we performed paired *t*-test to compare the EMG activity measured at each force level.

#### Intertrial Variability in Digit Placement

Although our experiment was designed to rule out differences in planning of digit position on each trial, it was important to confirm whether our design resulted in no difference in variability in digit contact points between 30% and 5% of force and between various TMS time points. To quantitatively evaluate this condition, we calculated the centre of pressure (CoP) for the thumb and index finger defined as the vertical and horizontal coordinates of the point of resultant force exerted by each digit on graspable surfaces of the grip device (Parikh and Cole, 2012).

(2) CoP=verticalMx-(Fy×w)Fn

(3) CoP=horizontalMy-(Fx×w)Fn

*M*_*x*_ and *M*_*y*_ are the moment about the *x*-axis and *y*-axis, respectively. *F*_*x*_ and *F*_*y*_ are the forces exerted on the grasp surface along the *x*-axis and *y*-axis, respectively. *w* is the distance between the surfaces of the F/T transducer and the grasp surface. *F*_*n*_ is the force component perpendicular to the grasp surface. To assess trial-to-trial variability in thumb and index finger CoPs, we computed area of an ellipse fitted to CoP (vertical and horizontal components calculated using Eqs. 2 and 3). For each subject, we calculated an ellipse that contained CoP points within 95% confidence interval in each force level and at each TMS time point, separately for thumb and index finger. Surface area of these ellipses gave us a measure of intertrial variability in CoP across trials at a given TMS time point and at a given force level, as established in previous studies (Duarte and Zatsiorsky, 2002; Davare et al., 2007; Friendly et al., 2013). We performed separate rmANOVA with within-subject factors such as FORCE (two levels: 30% and 5%) and TMS (six levels: 0.5, 0.75, 1, 1.1, 1.2, and 1.3 s) for the thumb and index finger.

For all statistical analyses involving rmANOVA (α = 0.05), we used Mauchly’s test to assess the assumption of sphericity and applied Greenhouse-Geisser correction when needed. *Post hoc* paired *t*-test comparisons were performed between adjacent TMS time points using Dunn-Sidak corrections. As stated earlier, we hypothesized that the modulation in CV of MEP and its relation with behavioral variability would be dependent on the magnitude of force (Dettmers et al., 1996; Ehrsson et al., 2001; Hendrix et al., 2009; Perez and Cohen, 2009; Parikh et al., 2014). Therefore, at each force level, we studied the modulation in CV of MEP using additional paired *t*-tests with appropriate Dunn-Sidak corrections and performed separate correlation analysis. The statistical analyses were performed using SPSS (version 25, SPSS Inc., Chicago, IL, United States).

## Results

### Intertrial Variability in Behavioral Measures

#### Variability in Time to Peak Force Rate (Time_PFR_)

**FIGURE 2:**
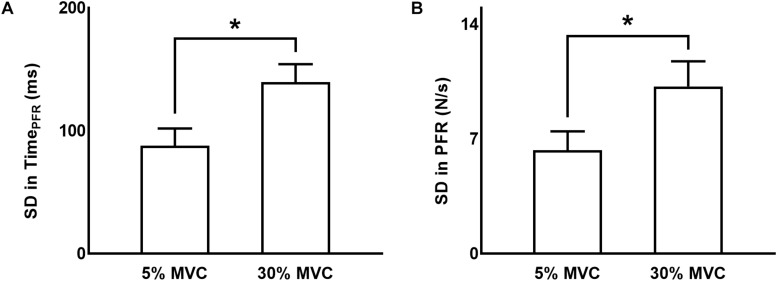
Variability in grip force rate variables. **(A,B)** Standard deviation (SD) in time to peak force rate and peak force rate, respectively, at 5% and 30% of force. Data are averages across all subjects (vertical bars denote SE). Asterisks indicate *p* < 0.05.

Standard deviation in Time_PFR_ from trial-to-trial was greater at 30% than 5% of force (main effect of FORCE: *F*_(1,11)_ = 31.160, *p* < 0.001, η_*p*_^2^ = 0.739; Figure 2A). We observed no modulation in variability in Time_PFR_ across different TMS delivery time points for TMS pulses (neither a main effect of TMS: *F*_(5,55)_ = 1.370, *p* = 0.250, η*_*p*_*^2^ = 0.111, nor FORCE × TMS interaction: *F*_(5,5)_ = 0.469, *p* = 0.660, η*_*p*_*^2^ = 0.041).

#### Variability in Magnitude of Peak Force Rate (PFR)

The standard deviation (SD) of PFR was greater at 30% than 5% of force (main effect of FORCE: *F*_(1,11)_ = 26.732, *p* < 0.001, η*_*p*_*^2^ = 0.708; Figure 2B). The delivery of TMS pulses at different time points did not influence the variability in PFR (neither a main effect of TMS: *F*_(5,55)_ = 0.244, *p* = 0.787, η*_*p*_*^2^ = 0.022, nor FORCE × TMS interaction: *F*_(5,55)_ = 2.456, *p* = 0.089, η*_*p*_*^2^ = 0.183).

#### Variability in Digit Contact Points (CoP)

**FIGURE 3:**
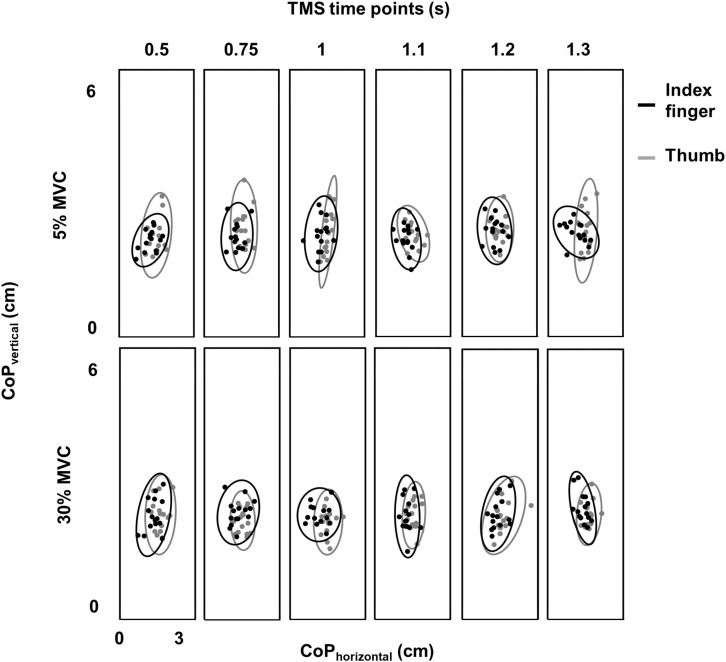
Variability in digit placement. Center of pressure (CoP) for thumb (gray) and index finger (black) for each TMS time point at 30% and 5% of force from a representative subject. Vertical and horizontal components of thumb and index finger CoP are shown on the same plot. Ellipse contained CoP points within 95% confidence interval in each task and at each TMS time point.

Variability in CoP was assessed by calculating the area of 95% confidence interval ellipse containing the digit contact points (Duarte and Zatsiorsky, 2002; Davare et al., 2007; Friendly et al., 2013). For the index finger contact point, we did not find difference in ellipse area between 30% and 5% of force across TMS time points (no main effect of FORCE: *F*_(1,11)_ = 0.38, *p* = 0.55, η*_*p*_*^2^ = 0.034; no main effect of TMS: *F*_(5,55)_ = 0.562, *p* = 0.728, η*_*p*_*^2^ = 0.049; no FORCE × TMS interaction: *F*_(5,55)_ = 0.606; *p* = 0.695, η*_*p*_*^2^ = 0.052; mean ± SE at 30% = 2.75 ± 0.19 cm^2^ and 5% = 2.71 ± 0.23 cm^2^; Figure 3). Similarly, for the thumb contact point, we did not find difference in ellipse area between 30% and 5% trials across TMS time points (30% = 3.67 ± 0.41 cm^2^ and 5% = 3.75 ± 0.40 cm^2^− no FORCE × TMS interaction: *F*_(5,55)_ = 0.58; *p* = 0.71, η*_*p*_*^2^ = 0.05; no main effect of FORCE: *F*_(1,11)_ = 0.11, *p* = 0.75, η*_*p*_*^2^ = 0.01; no main effect of TMS time points: *F*_(5,55)_ = 0.976, *p* = 0.44, η*_*p*_*^2^ = 0.08). These results suggest that the intertrial variability in digit contact points was similar across force levels and TMS time points.

### Relation Between MEP CV With MEP Amplitude at Rest

We modeled a relation between CV of MEP and amplitude of MEP separately for each muscle (FDI, APB, and ADM). The logarithmic relationship, as described in Eq. 1, for FDI was as below:

(4) CV=[(-0.1078)×log⁡(amplitude)]+0.5258

**FIGURE 4:**
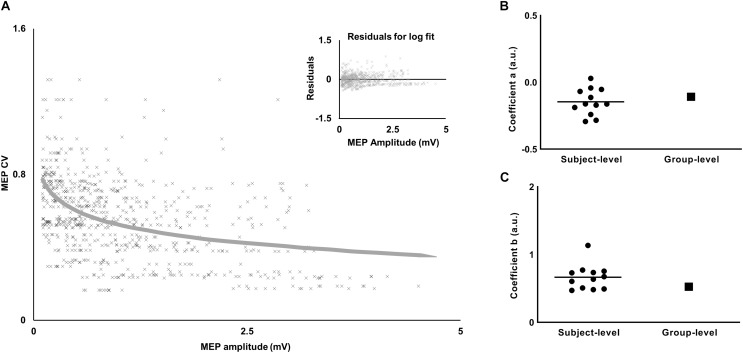
Relationship between variability (CV) and amplitude of MEP. **(A)** The decrease in MEP CV with increase in MEP amplitude was characterized by a logarithmic fit. Inset plot shows a trend in residuals for the logarithmic fit. **(B)** Comparison of the slope-coefficient for subject-level versus group-level models. **(C)** Comparison of the intercept-coefficient for subject-level versus group-level models. For the subject-level model, each dot represents coefficient from an individual subject and the horizontal line represents the mean.

The values of the coefficients (a, b) from equation 1 for APB were (−0.0899, 0.4764) and for ADM were (−0.0773, 0.3116). The logarithmic fit and the residuals for FDI are shown in Figure 4A.

### Variability in MEP Due to Changes in MEP Amplitude (CV_PRED_)

**FIGURE 5:**
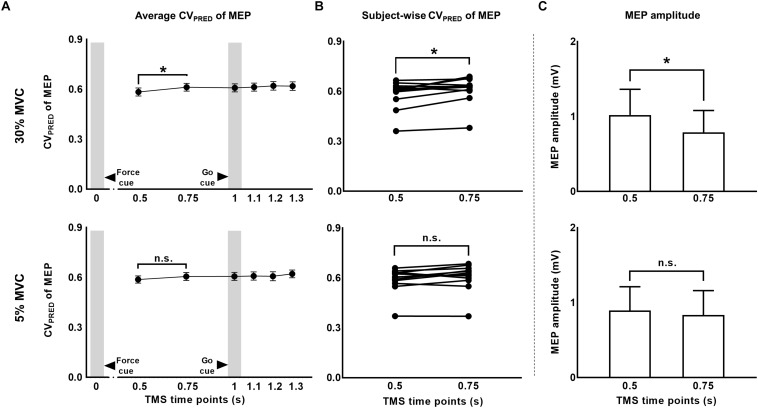
The CV of motor evoked potentials (MEP) due to changes in MEP amplitude. **(A)** Time-course of predicted CV of MEP at 30% compared to 5% of force. **(B)** Subject-wise predicted CV of MEP data indicates a consistent rise across subjects from 0.5 to 0.75 s at 30%, but not at 5% of force. **(C)** A significant reduction in MEP amplitude from 0.5 to 0.75 s explained the rise in predicted CV of MEP at 30% of force. Data in **(A,C)** are averages of all subjects (vertical bars denote SE). Asterisks indicate *p* < 0.016 and n.s. indicates *p* > 0.05.

We predicted MEP variability (CV_PRED_) for individual subjects using the logarithmic model separately for each muscle (Eqs. 1 and 4). For FDI, we found that CV_PRED_ of MEP was different across TMS time points (main effect of TMS: *F*_(5,55)_ = 3.695, *p* = 0.006, η*_*p*_*^2^ = 0.251; Figure 5A). However, this time-dependent modulation of CV_PRED_ of MEP was not different across force conditions (No FORCE × TMS interaction: *F*_(5,55)_ = 0.506, *p* = 0.770, η*_*p*_*^2^ = 0.044; no main effect of FORCE: *F*_(1,11)_ = 0.701, *p* = 0.420, η*_*p*_*^2^ = 0.060). *Post hoc* comparisons found a significant increase in CV_PRED_ of MEP from 1.2 to 1.3 s (*t*_11_ = 3.2, *p* = 0.009, Cohen’s *d*_Z_ = 0.92). No difference in CV_PRED_ of MEP was found between other adjacent TMS time points (all *p* values > 0.05). Within each force level, we found significant increase in CV_PRED_ of MEP from 0.5 to 0.75 s at 30% (*t*_11_ = 2.9, *p* = 0.015, Cohen’s *d*_Z_ = 0.84; Figures 5A,B), but not at 5% of force (*t*_11_ = 2.1, *p* = 0.06, Cohen’s *d*_Z_ = 0.61). The change in CV_PRED_ of MEP at 30% of force was related to the change in MEP amplitude. Specifically, we found a decrease in MEP amplitude for FDI from 0.5 to 0.75 s at 30% (*t*_11_ = 2.9, *p* = 0.014, Cohen’s *d*_Z_ = 0.84), but not at 5% (*t*_11_ = 1.4, *p* = 0.2, Cohen’s *d*_Z_ = 0.40) of force (Figure 5C).

For APB, we did not observe modulation in CV_PRED_ of MEP across force conditions and TMS time points (Table 1; No FORCE × TMS interaction: *F*_(5,45)_ = 0.588, *p* = 0.709, η*_*p*_*^2^ = 0.061; no main effect of FORCE: *F*_(1,9)_ = 2.313, *p* = 0.163, η*_*p*_*^2^ = 0.204; and no main effect of TMS: *F*_(5,45)_ = 0.988, *p* = 0.436, η*_*p*_*^2^ = 0.099). Similarly, for ADM, there was no modulation in predicted CV of MEP across force conditions and TMS time points (Table 1; No FORCE × TMS interaction: *F*_(5,35)_ = 0.724, *p* = 0.610, η*_*p*_*^2^ = 0.094; no main effect of FORCE: *F*_(1,7)_ = 0.841, *p* = 0.390, η*_*p*_*^2^ = 0.107; and no main effect of TMS: *F*_(5,35)_ = 0.090, *p* = 0.993, η*_*p*_*^2^ = 0.013).

**TABLE 1:** Predicted CV (CV_PRED_) of MEP for each TMS time point and force level.

Predicted CV of MEP												
TMS time point (s)	FDI				APB				ADM			
	5% MVC		30% MVC		5% MVC		30% MVC		5% MVC		30% MVC	
	Mean	SD	Mean	SD	Mean	SD	Mean	SD	Mean	SD	Mean	SD
0.5	0.588	0.076	0.584	0.084	0.506	0.055	0.506	0.063	0.416	0.058	0.401	0.059
0.75	0.606	0.084	0.613	0.081	0.526	0.054	0.512	0.043	0.415	0.057	0.406	0.056
1	0.607	0.085	0.609	0.085	0.528	0.056	0.521	0.070	0.406	0.066	0.408	0.057
1.1	0.609	0.087	0.613	0.085	0.511	0.049	0.523	0.065	0.406	0.058	0.403	0.064
1.2	0.607	0.093	0.621	0.088	0.513	0.055	0.504	0.044	0.411	0.057	0.406	0.060
1.3	0.621	0.082	0.619	0.088	0.512	0.062	0.512	0.058	0.411	0.064	0.408	0.059

### MEP Variability Above and Beyond Predicted CV of MEP (CV_DIFF_)

**FIGURE 6:**
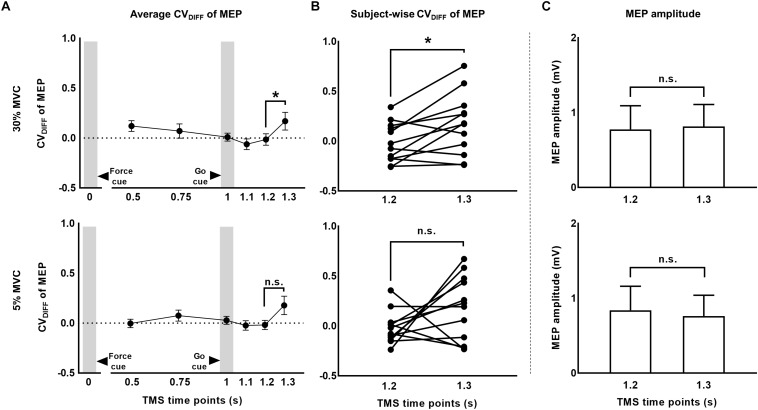
The CV of MEP rose above and beyond changes in MEP amplitude. **(A)** Time-course of CV_DIFF_ (observed – predicted CV) of MEP at 30% and 5% of force. **(B)** Subject-wise CV of MEP data indicates a consistent rise across subjects from 1.2 to 1.3 s at 30%, but not at 5% of force. **(C)** MEP amplitude analysis showed no modulation from 1.2 to 1.3 s. Data in **(A,C)** are averages of all subjects (vertical bars denote SE). Asterisks indicate *p* < 0.016 and n.s. indicates *p* > 0.1.

To investigate whether MEP variability modulated beyond CV_PRED_ of MEP during the force task, we subtracted CV_PRED_ of MEP (Table 1) from CV_OBS_ of MEP (Table 2) to obtain CV_DIFF_ of MEP (Table 3). For FDI, we found modulation in CV_DIFF_ of MEP across TMS time points (main effect of TMS: *F*_(5,55)_ = 4.730, *p* = 0.001, η*_*p*_*^2^ = 0.301). However, CV_DIFF_ of MEP was similar across force conditions (no FORCE × TMS interaction: *F*_(5,55)_ = 0.436, *p* = 0.821, η*_*p*_*^2^ = 0.038 and no main effect of FORCE: *F*_(1,11)_ = 0.065, *p* = 0.803, η*_*p*_*^2^ = 0.006). *Post hoc* comparisons found a significant increase in CV_DIFF_ of MEP from 1.2 to 1.3 s (*t*_11_ = 3.1, *p* = 0.01, Cohen’s *d*_Z_ = 0.89). No difference in CV_DIFF_ of MEP was found between other adjacent TMS time points (all *p* values > 0.13). Within each force level, we found significant increase in CV_DIFF_ of MEP from 1.2 to 1.3 s at 30% (*t*_11_ = 2.9, *p* = 0.015, Cohen’s *d*_Z_ = 0.84; Figure 6A) but not at 5% (*t*_11_ = 1.6, *p* = 0.14, Cohen’s *d*_Z_ = 0.46) of force. Most subjects showed a systematic increase in CV_DIFF_ of MEP from 1.2 s compared with 1.3 s at 30% of force (9 of 12 subjects; Figure 6B). However, at 5% of force, the change in CV_DIFF_ of MEP from 1.2 to 1.3 s was not consistent across subjects. Although the variability related to MEP amplitude were removed to obtain CV_DIFF_ of MEP, we confirmed that there was no change in MEP amplitude from 1.2 to 1.3 s (30%: *t*_11_ = 0.76, *p* = 0.47, Cohen’s *d*_Z_ = 0.22; 5%: *t*_11_ = 1.04, *p* = 0.32, Cohen’s *d*_Z_ = 0.30; Figure 6C). The increase in CV_DIFF_ of MEP was observed despite no change in background FDI activity (100 ms time window before TMS pulse) across TMS time points and force conditions. Specifically, we found that the intertrial variability (SD) of background FDI activity did not differ across TMS time points and force conditions (no main effect of TMS: *F*_(__5_,_55__)_ = 1.02, *p* = 0.42, η*_*p*_*^2^ = 0.08; no FORCE × TMS interaction: *F*_(__5_,_55__)_ = 0.74, *p* = 0.59, η*_*p*_*^2^ = 0.06, and no main effect of FORCE: *F*_(__1_,_11__)_ = 2.2, *p* = 0.16, η*_*p*_*^2^ = 0.17). Background EMG variability as assessed by CV yielded similar findings, i.e., no modulation with TMS time points or force

**TABLE 2:** Observed CV (CV_OBS_) of MEP for each TMS time point and force level.

Observed CV of MEP												
TMS time point (s)	FDI				APB				ADM			
	5% MVC		30% MVC		5% MVC		30% MVC		5% MVC		30% MVC	
	Mean	SD	Mean	SD	Mean	SD	Mean	SD	Mean	SD	Mean	SD
0.5	0.585	0.177	0.705	0.256	0.540	0.195	0.546	0.187	0.395	0.158	0.378	0.210
0.75	0.681	0.249	0.683	0.273	0.628	0.281	0.706	0.276	0.500	0.322	0.495	0.331
1	0.635	0.163	0.618	0.174	0.577	0.222	0.499	0.211	0.421	0.189	0.594	0.287
1.1	0.586	0.184	0.551	0.197	0.617	0.273	0.523	0.246	0.478	0.176	0.428	0.191
1.2	0.589	0.202	0.606	0.206	0.537	0.184	0.551	0.206	0.472	0.296	0.462	0.274
1.3	0.799	0.345	0.789	0.327	0.529	0.194	0.548	0.189	0.473	0.296	0.459	0.185

**TABLE 3:** Difference CV (CV_DIFF_) of MEP for each TMS time point and force level.

Difference CV of MEP												
TMS time point (s)	FDI				APB				ADM			
	5% MVC		30% MVC		5% MVC		30% MVC		5% MVC		30% MVC	
	Mean	SD	Mean	SD	Mean	SD	Mean	SD	Mean	SD	Mean	SD
0.5	−0.003	0.146	0.121	0.186	0.033	0.196	0.040	0.172	−0.021	0.162	−0.022	0.230
0.75	0.076	0.192	0.070	0.249	0.102	0.267	0.193	0.263	0.086	0.335	0.089	0.336
1	0.028	0.137	0.009	0.142	0.049	0.222	−0.022	0.238	0.015	0.194	0.186	0.271
1.1	−0.023	0.166	−0.062	0.189	0.106	0.267	0.000	0.267	0.072	0.144	0.025	0.186
1.2	−0.018	0.163	−0.014	0.198	0.025	0.189	0.046	0.196	0.061	0.291	0.056	0.279
1.3	0.178	0.323	0.170	0.308	0.017	0.187	0.036	0.183	0.061	0.303	0.050	0.191

conditions (no main effect of TMS: *F*_(__5_,_55__)_ = 1.37, *p* = 0.24, η*_*p*_*^2^ = 0.11; no FORCE × TMS interaction: *F*_(__5_,_55__)_ = 0.57, *p* = 0.72, η*_*p*_*^2^ = 0.04, and no main effect of FORCE: *F*_(__1_,_11__)_ = 2.27, *p* = 0.16, η*_*p*_*^2^ = 0.17). Similarly, the amplitude (RMS) of background EMG did not differ across TMS time points and force conditions (no main effect of TMS: *F*_(__5_,_55__)_ = 1.85, *p* = 0.12, η*_*p*_*^2^ = 0.14; no FORCE × TMS interaction: *F*_(__5_,_55__)_ = 2.01, *p* = 0.1, η*_*p*_*^2^ = 0.15, and no main effect of FORCE: *F*_(__1_,_11__)_ = 2.85 *p* = 0.12, η*_*p*_*^2^ = 0.2).

For APB, CV_DIFF_ of MEP was not different across force conditions and TMS time points (Table 3; no FORCE × TMS time points interaction: *F*_(__5_,_45__)_ = 0.302, *p* = 0.909, η*_*p*_*^2^ = 0.032; no main effect of TMS: *F*_(__5_,_45__)_ = 1.953, *p* = 0.104, η*_*p*_*^2^ = 0.178, and no main effect of Force: *F*_(__1_,_9__)_ = 0.290, *p* = 0.603, η*_*p*_*^2^ = 0.031). Similarly, for ADM, CV_DIFF_ of MEP was not different across force conditions and TMS time points (Table 3; no FORCE × TMS interaction: *F*_(__5_,_35__)_ = 0.746, *p* = 0.532, η*_*p*_*^2^ = 0.096; no main effect of TMS: *F*_(__5_,_35__)_ = 2.880, *p* = 0.073, η*_*p*_*^2^ = 0.118, and no main effect of FORCE: *F*_(__1_,_7__)_ = 0.938, *p* = 0.365, η*_*p*_*^2^ = 0.118).

**FIGURE 7:**
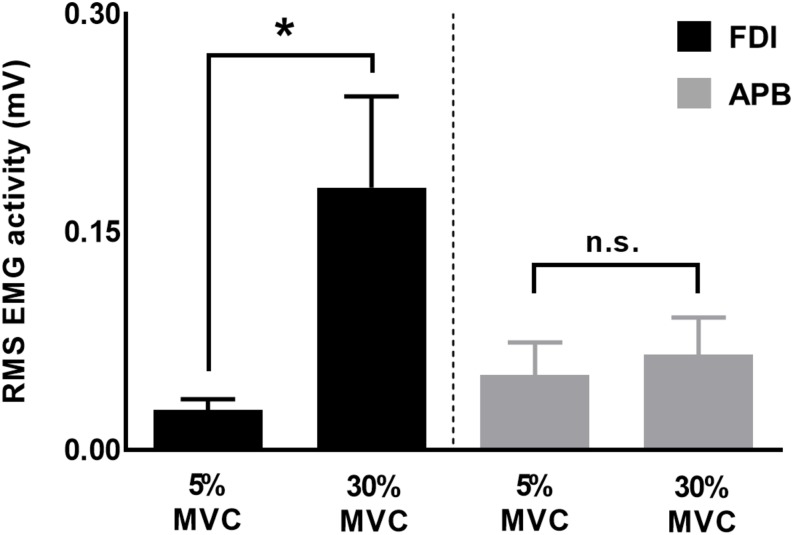
EMG activity for FDI and APB muscles. Force magnitude- dependent modulation in EMG activity was significant for FDI but not for APB muscles. Data are averages of all subjects (vertical bars denote SE), asterisk indicates *p* = 0.019 and n.s. indicates *p* > 0.1.

To understand muscle-specific modulation in CV_PRED_ and CV_DIFF_ of MEP, we investigated modulation in FDI and APB EMG activity at 30% and 5% of force. We found that the EMG activity was greater for 30% versus 5% of force for FDI (*t*_11_ = 2.7, *p* = 0.019, Cohen’s *d*_Z_ = 0.78), but not for APB (*t*_11_ = 1.4, *p* = 0.18, Cohen’s *d*_Z_ = 0.40; Figure 7), thus suggesting asymmetrical contribution of FDI and APB in the application of grip force, in agreement with previous reports (Li et al., 2013, 2015; Nataraj et al., 2015).

### Correlation Between the Rise in CV of MEP and Behavioral Variability

**FIGURE 8:**
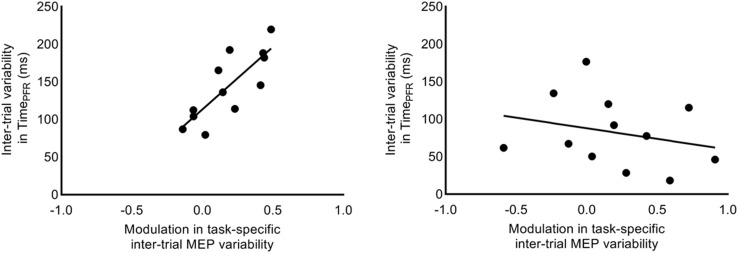
Correlation between intertrial task-specific variability in MEP and time to peak force rate. Modulation in intertrial MEP variability (CV_DIFF_ of MEP) for FDI muscle explained inter-individual differences in trial-to-trial fluctuations in time to peak force rate, selectively at 30% (*r* = 0.80, *p* = 0.0017), but not at 5% (*r* = –0.25, *p* = 0.4228) of force.

We investigated whether the increase in task-related MEP variability (i.e., CV_DIFF_ of MEP) from 1.2 to 1.3 s explained the inter-individual differences in trial-to-trial behavioral variability. We found that the increase in CV_DIFF_ of MEP from 1.2 to 1.3 s explained 64% of inter-individual differences in SD of Time_PFR_ (Pearson’s *r* = 0.80, *p* = 0.0017; Figure 8) at 30% of force. However, similar association between CV_DIFF_ of MEP and SD in Time_PFR_ was not observed for 5% of force (*r* = −0.25, *p* = 0.42). Similarly, the increase in SD_DIFF_ of MEP from 1.2 to 1.3 s explained 39% of inter-individual differences in SD of Time_PFR_ (Pearson’s *r* = 0.62; *p* = 0.03) at 30% of force, but not at 5% of force (Pearson’s *r* = −0.20; *p* = 0.53). We found no correlation between CV_DIFF_ of MEP and SD of PFR or CoP variability (all *r*-values < 0.26, all *p* values > 0.42).

### Robustness and Repeatability of the Findings

To test the robustness of the findings with respect to MEP pre-processing, we analyzed our data using no lower bound cut-off for MEP amplitude and no bin-based cut-off criteria (see section “Materials and Methods”). Furthermore, we fitted a logarithmic model for each individual subject’s data to understand if the findings were sensitive to the group-level model (see section “Materials and Methods”). The results were similar to that reported above. Across individual logarithmic models, the intercept ranged from 0.47 to 1.13 and the slope ranged from −0.29 to 0.029 (Figures 4B,C). The logarithmic model obtained from *experiment 1* was used to obtain CV_PRED_ for each subject. As done earlier, we subtracted CV_PRED_ of MEP from CV_OBS_ of MEP to obtain CV_DIFF_ of MEP. We found modulation in CV_DIFF_ of MEP in FDI across TMS time points (main effect of TMS: *F*_(__5_,_55__)_ = 7.64, *p* < 0.001, η*_*p*_*^2^ = 0.41). CV_DIFF_ of MEP was similar across force conditions (no FORCE × TMS interaction: *F*_(__5_,_55__)_ = 0.55, *p* = 0.73, η*_*p*_*^2^ = 0.048 and no main effect of FORCE: *F*_(__1_,_11__)_ = 0.021, *p* = 0.88, η*_*p*_*^2^ = 0.002). *Post hoc* comparisons found a significant increase in CV_DIFF_ of MEP from 1.2 to 1.3 s (*t*_11_ = 3.92, *p* = 0.002, Cohen’s *d*_Z_ = 1.13). No difference in CV_DIFF_ of MEP was found between other adjacent TMS time points (all *p* values > 0.10). Within each force level, we found significant increase in CV_DIFF_ of MEP from 1.2 to 1.3 s at 30% (*t*_11_ = 2.9, *p* = 0.015, Cohen’s *d*_Z_ = 0.84) but not at 5% (*t*_11_ = 1.9, *p* = 0.08, Cohen’s d*_Z_* = 0.55) of force. Importantly, the relationship between the modulation in CV_DIFF_ of MEP and intertrial behavioral variability was preserved. That is, the increase in CV_DIFF_ of MEP from 1.2 to 1.3 s explained 61% of inter-individual differences in Time_PFR_ SD (Pearson’s *r* = 0.77, *p* = 0.0029) at 30% of force.

To test the repeatability of the MEP findings, we separately analyzed data from nine additional subjects who had performed a similar task (low force = 1 N grasp force and high force = 10% of force) as described in Parikh et al. (2014). We found modulation in CV_DIFF_ of MEP in FDI across TMS time points (main effect of TMS: *F*_(__5_,_40__)_ = 3.63, *p* = 0.0081, η*_*p*_*^2^ = 0.31). CV_DIFF_ of MEP was similar across force conditions (no FORCE × TMS interaction: *F*_(__5_,_40__)_ = 0.41, *p* = 0.84, η*_*p*_*^2^ = 0.048 and no main effect of FORCE: *F*_(__1_,_8__)_ = 0.029, *p* = 0.86, η*_*p*_*^2^ = 0.004). *Post hoc* comparisons found an increase in CV_DIFF_ of MEP from 1.2 to 1.3 s (*t*_8_ = 2.61, *p* = 0.03, Cohen’s *d*_z_ = 0.87), however, it failed to reach the corrected significance level likely due to lower sample size than the main study. No difference in CV_DIFF_ of MEP was found between other adjacent TMS time points (all *p* values > 0.15). Within each force level, we found an increase (although non-significant potentially due to low sample size) in CV_DIFF_ of MEP from 1.2 to 1.3 s for the high force condition (10% of force; *t*_8_ = 2.16, *p* = 0.06, Cohen’s *d*_Z_ = 0.72) but not for the low force condition (1 N grasp force; *t*_8_ = 1.8, *p* = 0.09, Cohen’s *d*_Z_ = 0.60). We found that the increase in CV_DIFF_ of MEP from 1.2 to 1.3 s explained 40% of inter-individual differences in SD of Time_PFR_ for the high force condition (Pearson’s *r* = 0.63, *p* = 0.09). Similarly, the increase in SD_DIFF_ of MEP from 1.2 to 1.3 s explained 32% of inter-individual differences in SD of Time_PFR_ for the high force condition (Pearson’s *r* = 0.56; *p* = 0.1). The findings were not significant likely due to lower sample size in the repeatability dataset. For the low force condition, the detection of the time to peak force rate was not reliable because, as instructed, subjects exerted minimal force (∼1 N) perpendicular to its gripping surfaces (Parikh et al., 2014).

## Discussion

We found that CSE variability increased beyond changes observed in CSE magnitude (i.e., CV_DIFF_ of MEP) prior to the performance of the self-paced reach-to-grasp task. The increase in CSE variability occurred after the “go” cue presentation and this effect was temporally dissociated from the decrease in CSE magnitude that occurred before the “go” cue presentation. The time-dependent modulation in CSE variability and CSE amplitude was evident at 30%, but not at 5% of force. Importantly, at 30% of force, individuals with larger increase in CSE variability also exhibited larger intertrial variability in time to peak force rate. These results were found to be repeatable across studies and robust to different data-analysis methods. We discuss our findings in relation to potential sources underlying the increase in CSE variability and its contribution to the application of grip force.

### Modulation in CSE Variability

Using a logarithmic model relating CSE magnitude and variability, we predicted the component of variability in CSE during the task that can be attributed to changes in CSE magnitude. We found a significant increase in predicted CV of MEP at 30%, but not at 5% of force. As predicted CV is primarily influenced by CSE magnitude, we found a corresponding reduction in CSE magnitude from 0.5 to 0.75 s following the “force” cue presentation at 30%, but not at 5% of force. This finding is consistent with our previous report demonstrating modulation in CSE magnitude at a higher force (Parikh et al., 2014). In contrast, other studies have reported an increase in MEP amplitude prior to movement onset (Starr et al., 1988; Chen et al., 1998; Chen and Hallett, 1999). However, this discrepancy might be due to differences in the task requirements. For instance, Chen et al. (1998) used a self-paced task and in the current study we used an externally cued task consisting of a multi-joint precision grip characterized by contact forces. We further show that the intertrial variability in CSE rose beyond predicted variability in CSE at 30%, but not at 5% of force. Interestingly, the decrease in CSE magnitude and the increase in task-specific variability in CSE were temporally dissociated because the later occurred from 1.2 to 1.3 s following the presentation of “force” cue (i.e., after the “go” cue). These findings suggest distinct neural sources underlying the modulation in CSE magnitude and the component of CSE variability not related to changes in its magnitude. It is plausible that the reduction in MEP size following the “force” cue presentation represents digit force planning (Parikh et al., 2014) while the increase in MEP variability might represent retrieval of memory related to task-specific characteristics or features based on the presentation of the anticipated “go” cue (Singhal et al., 2013). We cannot rule out a possibility that the motor plan is processed in the time between the “force” cue and the “go” cue but is not processed after the “go” cue and until movement onset. Moreover, a consistent change in CSE magnitude and variability measures across individuals at 30% of force (Figures 5B, 6B) might represent important characteristics of individuals and thus the modulation in neural underpinnings prior to the onset of reach (Kanai and Rees, 2011). Functional magnetic resonance imaging work has shown stronger activation in sensory- and motor-related fronto-parietal brain areas during application of smaller compared to larger precision grip force (Ehrsson et al., 2001). These findings suggest that precision grasping using smaller forces is a function of activation within a wider brain network. It is plausible that similar force-dependent activation is also present prior to the onset of reach (Hendrix et al., 2009). Large between-subject differences in the activation patterns within this wider network might have contributed to inconsistent modulation of MEP variability prior to the onset of reach at 5% of force. Lower and focal brain activation for 30% of force might have led to more consistent modulation of MEP variability.

### Potential Mechanisms That Increased CSE Variability

Our experimental design ruled out any difference in planning of digit position from trial-to-trial between force levels and across time points. These findings may suggest that the modulation in CSE variability was specific to the task of grip force application.

Corticospinal excitability arises from activation of intracortical circuitry within M1, cortico-cortical inputs to M1, and subcortical and spinal structures (Bestmann and Krakauer, 2015). The modulation in neuronal activity within primate M1 and premotor cortices has been found to depend on the magnitude of grasp force (Hendrix et al., 2009). Parietal, occipital, cerebellum, dorsolateral prefrontal cortex, and basal ganglia are also known to contribute to the planning of grip force (Dettmers et al., 1996; Ehrsson et al., 2000, 2001; Chouinard et al., 2005; Berner et al., 2007; Davare et al., 2007; Dafotakis et al., 2008). Virtual lesion studies using TMS have demonstrated contribution of human somatosensory, premotor dorsal, and supplementary motor regions in regulating the timing of digit force application (Davare et al., 2006; Schabrun et al., 2008; White et al., 2013). Evidence also exists in humans about the functional role of reticulospinal tracts in the control of coordinated hand movements such as those performed in our study (Honeycutt et al., 2013). It is less likely that changes in spinal motor neuron pool directly contributed to the increase in CSE variability because variability in spinal motor neuronal excitability (as assessed by modulation in H-reflex) has been suggested to arise from changes in descending drive from supraspinal structures to spinal cord during motor planning (Collins et al., 1993; Misiaszek, 2003). Taken together, the increase in CSE variability observed prior to the onset of reach in our study is potentially sourced within supraspinal structures. Modulation in activation of these potential sources might have contributed to intertrial fluctuations in presynaptic inputs to M1 neurons (Lemon, 2008), thus resulting in modulation in CSE variability. As noted above, the inputs to M1 that influence CSE magnitude (Parikh et al., 2014) might be distinct from the inputs to M1 that influence CSE variability.

### Rise in CSE Variability Explains Inter-Individual Differences in Behavioral Variability

In monkeys, neuronal firing rate variability within M1 and premotor regions prior to movement onset has been suggested to explain ∼50% of variability in reach speed from trial-to-trial (Churchland et al., 2006a, b). Consistent with this primate work, we found that the modulation in intertrial variability in CSE prior to movement onset explained at least ∼40% of inter-individual differences in behavioral, viz. Time_PFR_, variability in humans. The rise in CSE variability was associated with Time_PFR_ variability but not with variability in magnitude of peak force rate, although both factors are known to be important for accurate force application (Poston et al., 2008). It is plausible that the intertrial variability in CSE may encode the variability in timing of force application as a control variable. Disruption of human premotor dorsal area using single pulse TMS prior to grasp was found to affect the timing, but not the magnitude, of grip force application (Davare et al., 2006). The observed relationship between CSE and Time_PFR_ variability, therefore, may suggest the contribution of premotor dorsal area to the modulation in CSE variability. Interestingly, single pulse TMS over M1 as used in the current study did not impair subjects’ ability to control digit placement and apply grip force. Further studies using repetitive TMS, a more robust way to perturb neural activity (Paus, 2005), might provide better insight into the central mechanisms underlying behavioral variability. Other task attributes such as attention and arousal levels may also contribute to behavioral and neural variability (Cohen et al., 1997; Fontanini and Katz, 2008; Masquelier, 2013; Dinstein et al., 2015). Our findings provide evidence for the contribution of variability in neural mechanisms prior to movement onset to motor output variability and corroborate earlier behavioral work in humans (van Beers, 2009). Fluctuations in neural activity during reach-to-grasp task performance may explain the remaining inter-individual differences in timing variability. A recent neuroimaging study found that the variability in BOLD-activity within intraparietal cortex recorded concurrently with task performance accounts for ∼25% of inter-individual differences in movement extent variability (Haar et al., 2017). In our study, the neural activity engaged prior to the onset of reach may also be present during task performance and thus potentially contributing to the inter-individual differences in behavioral variability.

Overall, our study provides a novel insight into the contribution of neural mechanisms prior to movement onset to behavioral variability by assessing variability in human CSE in a self-paced reach-to-grasp paradigm. Our findings suggest that individuals with a greater increase in the neural variability prior to reach onset exhibit greater behavioral variability.

## Data Availability Statement

All datasets generated for this study are included in the article/supplementary material.

## Ethics Statement

The studies involving human participants were reviewed and approved by the Institutional Review Board of the University of Houston. The patients/participants provided their written informed consent to participate in this study.

## Author Contributions

NR and PP designed the study and prepared the manuscript. NR performed data collection and data analysis. PP reviewed the data analysis.

## Conflict of Interest

The authors declare that the research was conducted in the absence of any commercial or financial relationships that could be construed as a potential conflict of interest.
